# (*E*)-Methyl 3-(2-methyl-1-phenyl­sulfonyl-1*H*-indol-3-yl)but-2-enoate

**DOI:** 10.1107/S1600536809002931

**Published:** 2009-01-31

**Authors:** T. Kavitha, M. Thenmozhi, G. Gobi Rajeshwaran, A. K. Mohanakrishnan, M. N. Ponnuswamy

**Affiliations:** a Centre of Advanced Study in Crystallography and Biophysics, University of Madras, Guindy Campus, Chennai 600 025, India; b Department of Organic Chemistry, University of Madras, Guindy Campus, Chennai 600 025, India

## Abstract

In the title compound, C_20_H_19_NO_4_S, the indole ring system is planar  [r.m.s. deviation = 0.023 (2) Å]. The sulfonyl-bound phenyl ring is almost perpendicular to the indole ring system [dihedral angle = 86.75 (7)°]. The ester group is almost planar (r.m.s. deviation = 0.030 Å) and is oriented at an angle of 62.53 (5)° with respect to the indole ring system. Mol­ecules are linked into a two-dimensional network parallel to the *ab* plane by inter­molecular C—H⋯O hydrogen bonds.

## Related literature

For the biological activities of indole and its derivatives, see: Chandrakantha *et al.* (1992 ▶); Rodriguez *et al.* (1985 ▶). For related literature For the configuration at the S atom, see: Bassindale (1984 ▶). For the N atom hybridization, see: Beddoes *et al.* (1986 ▶).
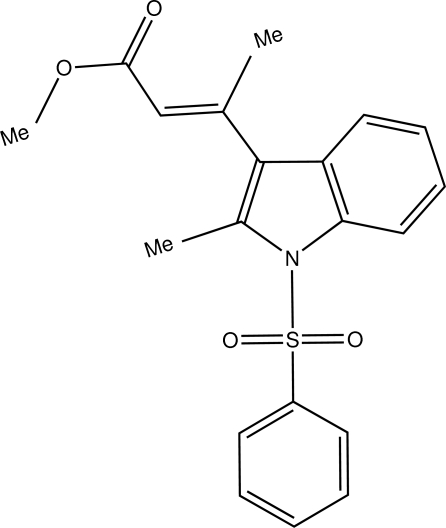


## Experimental

### 

#### Crystal data

C_20_H_19_NO_4_S
*M* *_r_* = 369.42
Monoclinic, 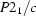

*a* = 8.9498 (3) Å
*b* = 8.8427 (2) Å
*c* = 23.2836 (7) Å
β = 97.085 (1)°
*V* = 1828.60 (9) Å^3^
*Z* = 4
Mo *K*α radiation
μ = 0.20 mm^−1^
*T* = 293 (2) K
0.25 × 0.20 × 0.16 mm

#### Data collection

Bruker APEXII CCD area-detector diffractometer
Absorption correction: multi-scan (*SADABS*; Sheldrick, 2001 ▶) *T*_min_ = 0.957, *T*_max_ = 0.968
23203 measured reflections
5673 independent reflections
3833 reflections with *I* > 2σ(*I*)
*R*_int_ = 0.026

#### Refinement

*R*[*F* ^2^ > 2σ(*F* ^2^)] = 0.046
*wR*(*F* ^2^) = 0.137
*S* = 1.01
5673 reflections
239 parameters
H-atom parameters constrained
Δρ_max_ = 0.30 e Å^−3^
Δρ_min_ = −0.29 e Å^−3^

### 

Data collection: *APEX2* (Bruker, 2004 ▶); cell refinement: *SAINT* (Bruker, 2004 ▶); data reduction: *SAINT*; program(s) used to solve structure: *SHELXS97* (Sheldrick, 2008 ▶); program(s) used to refine structure: *SHELXL97* (Sheldrick, 2008 ▶); molecular graphics: *ORTEP-3* (Farrugia, 1997 ▶); software used to prepare material for publication: *SHELXL97* and *PLATON* (Spek, 2003 ▶).

## Supplementary Material

Crystal structure: contains datablocks I, global. DOI: 10.1107/S1600536809002931/ci2752sup1.cif

Structure factors: contains datablocks I. DOI: 10.1107/S1600536809002931/ci2752Isup2.hkl

Additional supplementary materials:  crystallographic information; 3D view; checkCIF report

## Figures and Tables

**Table 1:** Hydrogen-bond geometry (Å, °)

*D*—H⋯*A*	*D*—H	H⋯*A*	*D*⋯*A*	*D*—H⋯*A*
C6—H6⋯O2^i^	0.93	2.58	3.391 (2)	146
C13—H13⋯O3^ii^	0.93	2.50	3.277 (2)	141

Symmetry codes: (i) 
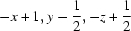
; (ii) 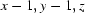.

**Fig. 1.**
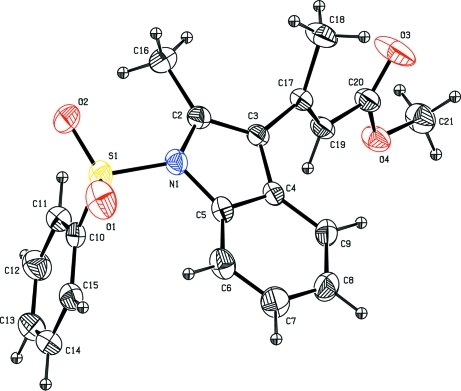
The molecular structure of the title compound. Displacement ellipsoids are drawn at the 20% probability level.

**Fig. 2.**
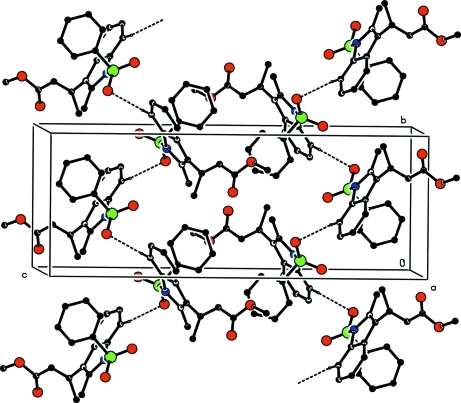
Crystal packing of the title compound, viewed down the a axis. H atoms not involved in hydrogen bonding have been omitted for clarity.

## supplementary crystallographic information

### Comment

Indole and its derivatives have long been known for their chemical and biological activities (Chandrakantha *et al.*, 1992). The indole ring system is present in a number of natural products, many of which are found to possess pharmacological properties like anti-microbial, anti-inflammatory and anti-implantation activities (Rodriguez *et al.*, 1985).

Due to Thorpe–Ignold effect (Bassindale, 1984), bond angles around atom S1 show significant deviation from ideal tetrahedral value, with significant deviations in angles O1—S1—O2 [120.37 (9)°] and N1—S1—C10 [104.96 (6)°]. The indole ring system is essentially planar. The sum of the bond angles around atom N1 (355.9°) indicates *sp*^2^ hybridization (Beddoes *et al.*, 1986). The sulfonyl bound phenyl ring is oriented almost perpendicular to the indole ring system as can be seen from the dihedral angle of 86.75 (7)°. The ester group attached to the indole ring system adopts an extended conformation which is confirmed by the torsion angles C3—C17—C19—C20 = -177.59 (14)°, C17—C19—C20—O4 = -176.02 (15)° and C19—C20—O4—C21 = -178.62 (15)°.

In the crystal structure, intermolecular C—H···O hydrogen bonds (Table 1) link the molecules into a two-dimensional network parallel to the *ab* plane (Fig. 2).

### Experimental

To a stirred suspension of NaH (29 mg, 1.20 mmol, hexane washed) in THF (5 ml), a solution of vinyl indole (0.23 g, 1 mmol) in THF (5 ml) was added and stirred for 30 min at room temperature. To the reaction mixture, a solution of PhSO_2_Cl (0.21 g, 1.20 mmol) was added and stirring was continued for further 6 h. After the indole was consumed (monitored by TLC), the reaction mixture was quenched with cold diluted HCl (25 ml), extracted with ethyl acetate (2 × 10 ml) and dried (Na_2_SO_4_). Removal of solvent followed by recrystallization (MeOH) afforded yellow crystals of the title compound.

### Refinement

H atoms were positioned geometrically (C—H = 0.93–0.96 Å) and allowed to ride on their parent atoms, with *U*_iso_(H) = 1.5*U*_eq_(C) for methyl H and 1.2*U*_eq_(C) for other H atoms.

### Crystal data

**Table:**

C_20_H_19_NO_4_S	*F*(000) = 776
*M**_r_* = 369.42	*D*_x_ = 1.342 Mg m^−^^3^
Monoclinic, *P*2_1_/*c*	Mo *K*α radiation, λ = 0.71073 Å
Hall symbol: -P 2ybc	Cell parameters from 5673 reflections
*a* = 8.9498 (3) Å	θ = 2.3–31.0°
*b* = 8.8427 (2) Å	µ = 0.20 mm^−^^1^
*c* = 23.2836 (7) Å	*T* = 293 K
β = 97.085 (1)°	Block, yellow
*V* = 1828.60 (9) Å^3^	0.25 × 0.20 × 0.16 mm
*Z* = 4	

### Data collection

**Table:**

Bruker APEXII CCD area-detector diffractometer	5673 independent reflections
Radiation source: fine-focus sealed tube	3833 reflections with *I* > 2σ(*I*)
graphite	*R*_int_ = 0.026
ω and φ scans	θ_max_ = 31.0°, θ_min_ = 2.3°
Absorption correction: multi-scan (*SADABS*; Sheldrick, 2001)	*h* = −12→11
*T*_min_ = 0.957, *T*_max_ = 0.968	*k* = −7→12
23203 measured reflections	*l* = −32→33

### Refinement

**Table:**

Refinement on *F*^2^	Secondary atom site location: difference Fourier map
Least-squares matrix: full	Hydrogen site location: inferred from neighbouring sites
*R*[*F*^2^ > 2σ(*F*^2^)] = 0.046	H-atom parameters constrained
*wR*(*F*^2^) = 0.137	*w* = 1/[σ^2^(*F*_o_^2^) + (0.0625*P*)^2^ + 0.402*P*] where *P* = (*F*_o_^2^ + 2*F*_c_^2^)/3
*S* = 1.01	(Δ/σ)_max_ = 0.016
5673 reflections	Δρ_max_ = 0.30 e Å^−^^3^
239 parameters	Δρ_min_ = −0.29 e Å^−^^3^
0 restraints	Extinction correction: *SHELXL97* (Sheldrick, 2008), Fc^*^=kFc[1+0.001xFc^2^λ^3^/sin(2θ)]^-1/4^
Primary atom site location: structure-invariant direct methods	Extinction coefficient: 0.0057 (11)

### Special details

**Table:**

Geometry. All e.s.d.'s (except the e.s.d. in the dihedral angle between two l.s. planes) are estimated using the full covariance matrix. The cell e.s.d.'s are taken into account individually in the estimation of e.s.d.'s in distances, angles and torsion angles; correlations between e.s.d.'s in cell parameters are only used when they are defined by crystal symmetry. An approximate (isotropic) treatment of cell e.s.d.'s is used for estimating e.s.d.'s involving l.s. planes.
Refinement. Refinement of *F*^2^ against ALL reflections. The weighted *R*-factor *wR* and goodness of fit *S* are based on *F*^2^, conventional *R*-factors *R* are based on *F*, with *F* set to zero for negative *F*^2^. The threshold expression of *F*^2^ > σ(*F*^2^) is used only for calculating *R*-factors(gt) *etc*. and is not relevant to the choice of reflections for refinement. *R*-factors based on *F*^2^ are statistically about twice as large as those based on *F*, and *R*- factors based on ALL data will be even larger.

### Fractional atomic coordinates and isotropic or equivalent isotropic displacement parameters (Å^2^)

**Table:**

	*x*	*y*	*z*	*U*_iso_*/*U*_eq_	
C2	0.71830 (17)	0.73053 (17)	0.13969 (7)	0.0461 (3)	
C3	0.85936 (15)	0.68775 (16)	0.13101 (6)	0.0406 (3)	
C4	0.89541 (15)	0.54808 (16)	0.16149 (6)	0.0408 (3)	
C5	0.77241 (15)	0.50989 (17)	0.19043 (6)	0.0430 (3)	
C6	0.77105 (19)	0.3804 (2)	0.22371 (7)	0.0587 (4)	
H6	0.6889	0.3562	0.2428	0.070*	
C7	0.8966 (2)	0.2887 (2)	0.22744 (8)	0.0703 (5)	
H7	0.8983	0.2001	0.2490	0.084*	
C8	1.0202 (2)	0.3256 (2)	0.19985 (9)	0.0682 (5)	
H8	1.1036	0.2621	0.2036	0.082*	
C9	1.02144 (17)	0.45440 (19)	0.16699 (7)	0.0546 (4)	
H9	1.1050	0.4786	0.1487	0.065*	
C10	0.42383 (15)	0.44900 (18)	0.13523 (7)	0.0462 (3)	
C11	0.38591 (18)	0.4876 (2)	0.07757 (7)	0.0550 (4)	
H11	0.3946	0.5871	0.0655	0.066*	
C12	0.3352 (2)	0.3766 (2)	0.03848 (9)	0.0702 (5)	
H12	0.3097	0.4009	−0.0003	0.084*	
C13	0.3223 (2)	0.2295 (2)	0.05676 (10)	0.0739 (6)	
H13	0.2870	0.1553	0.0302	0.089*	
C14	0.3607 (2)	0.1917 (2)	0.11349 (11)	0.0720 (5)	
H14	0.3524	0.0919	0.1253	0.086*	
C15	0.41202 (19)	0.3013 (2)	0.15350 (9)	0.0598 (4)	
H15	0.4382	0.2760	0.1922	0.072*	
C16	0.6333 (2)	0.8675 (2)	0.11697 (10)	0.0724 (5)	
H16A	0.6151	0.9315	0.1487	0.109*	
H16B	0.5389	0.8374	0.0960	0.109*	
H16C	0.6911	0.9218	0.0916	0.109*	
C17	0.96483 (16)	0.77322 (16)	0.09861 (6)	0.0430 (3)	
C18	1.0118 (2)	0.92731 (19)	0.12070 (8)	0.0648 (5)	
H18A	1.1128	0.9231	0.1400	0.097*	
H18B	0.9452	0.9609	0.1474	0.097*	
H18C	1.0074	0.9967	0.0888	0.097*	
C19	1.01726 (16)	0.70573 (17)	0.05409 (7)	0.0466 (3)	
H19	0.9820	0.6090	0.0444	0.056*	
C20	1.12631 (18)	0.77169 (18)	0.01894 (7)	0.0497 (4)	
C21	1.2556 (2)	0.7283 (2)	−0.06194 (8)	0.0670 (5)	
H21A	1.2073	0.7956	−0.0909	0.101*	
H21B	1.2938	0.6419	−0.0804	0.101*	
H21C	1.3373	0.7801	−0.0395	0.101*	
N1	0.66229 (13)	0.62376 (15)	0.17753 (5)	0.0465 (3)	
O1	0.48023 (16)	0.53321 (19)	0.24184 (5)	0.0801 (4)	
O2	0.40094 (14)	0.72716 (15)	0.16816 (7)	0.0756 (4)	
O3	1.1914 (2)	0.88897 (17)	0.02638 (7)	0.0984 (6)	
O4	1.14823 (13)	0.67953 (13)	−0.02447 (5)	0.0583 (3)	
S1	0.48136 (4)	0.59233 (5)	0.185184 (18)	0.05532 (15)	

### Atomic displacement parameters (Å^2^)

**Table:**

	*U*^11^	*U*^22^	*U*^33^	*U*^12^	*U*^13^	*U*^23^
C2	0.0440 (7)	0.0399 (7)	0.0552 (8)	−0.0028 (6)	0.0092 (6)	−0.0070 (6)
C3	0.0391 (7)	0.0365 (7)	0.0464 (7)	−0.0050 (6)	0.0066 (5)	−0.0042 (6)
C4	0.0347 (6)	0.0432 (7)	0.0444 (7)	−0.0066 (6)	0.0039 (5)	−0.0023 (6)
C5	0.0372 (7)	0.0527 (8)	0.0390 (7)	−0.0063 (6)	0.0038 (5)	−0.0022 (6)
C6	0.0520 (9)	0.0750 (12)	0.0489 (8)	−0.0117 (8)	0.0058 (7)	0.0168 (8)
C7	0.0648 (11)	0.0738 (13)	0.0695 (11)	−0.0025 (10)	−0.0021 (9)	0.0318 (10)
C8	0.0492 (9)	0.0664 (12)	0.0872 (13)	0.0085 (9)	0.0006 (9)	0.0222 (10)
C9	0.0373 (7)	0.0568 (10)	0.0698 (10)	0.0002 (7)	0.0074 (7)	0.0080 (8)
C10	0.0295 (6)	0.0496 (8)	0.0607 (9)	0.0002 (6)	0.0104 (6)	−0.0014 (7)
C11	0.0497 (9)	0.0484 (9)	0.0661 (10)	−0.0034 (7)	0.0034 (7)	0.0018 (7)
C12	0.0663 (12)	0.0699 (12)	0.0706 (12)	−0.0108 (10)	−0.0063 (9)	−0.0070 (9)
C13	0.0593 (11)	0.0595 (12)	0.0994 (15)	−0.0112 (9)	−0.0042 (10)	−0.0160 (11)
C14	0.0521 (10)	0.0459 (10)	0.1173 (17)	−0.0077 (8)	0.0082 (10)	0.0056 (10)
C15	0.0433 (8)	0.0582 (10)	0.0782 (11)	−0.0038 (7)	0.0079 (8)	0.0114 (9)
C16	0.0593 (11)	0.0506 (10)	0.1084 (16)	0.0094 (8)	0.0149 (10)	0.0063 (10)
C17	0.0406 (7)	0.0369 (7)	0.0511 (8)	−0.0059 (6)	0.0045 (6)	0.0012 (6)
C18	0.0794 (12)	0.0473 (9)	0.0714 (11)	−0.0209 (9)	0.0234 (9)	−0.0137 (8)
C19	0.0461 (8)	0.0376 (7)	0.0576 (8)	−0.0109 (6)	0.0119 (6)	−0.0019 (6)
C20	0.0513 (8)	0.0421 (8)	0.0574 (9)	−0.0096 (7)	0.0139 (7)	−0.0024 (7)
C21	0.0692 (11)	0.0700 (12)	0.0673 (11)	−0.0114 (9)	0.0306 (9)	−0.0010 (9)
N1	0.0389 (6)	0.0512 (7)	0.0510 (7)	−0.0031 (5)	0.0118 (5)	−0.0068 (5)
O1	0.0728 (8)	0.1164 (12)	0.0584 (7)	−0.0149 (8)	0.0369 (6)	−0.0148 (7)
O2	0.0534 (7)	0.0642 (8)	0.1135 (11)	0.0126 (6)	0.0278 (7)	−0.0269 (8)
O3	0.1299 (14)	0.0687 (9)	0.1106 (12)	−0.0562 (9)	0.0702 (10)	−0.0333 (8)
O4	0.0646 (7)	0.0522 (7)	0.0626 (7)	−0.0135 (6)	0.0255 (5)	−0.0074 (5)
S1	0.0418 (2)	0.0660 (3)	0.0624 (3)	−0.00185 (18)	0.02337 (17)	−0.01782 (19)

### Geometric parameters (Å, °)

**Table:**

C2—C3	1.357 (2)	C13—H13	0.93
C2—N1	1.4241 (19)	C14—C15	1.383 (3)
C2—C16	1.493 (2)	C14—H14	0.93
C3—C4	1.441 (2)	C15—H15	0.93
C3—C17	1.4857 (19)	C16—H16A	0.96
C4—C9	1.393 (2)	C16—H16B	0.96
C4—C5	1.4012 (19)	C16—H16C	0.96
C5—C6	1.383 (2)	C17—C19	1.331 (2)
C5—N1	1.415 (2)	C17—C18	1.498 (2)
C6—C7	1.380 (3)	C18—H18A	0.96
C6—H6	0.93	C18—H18B	0.96
C7—C8	1.385 (3)	C18—H18C	0.96
C7—H7	0.93	C19—C20	1.470 (2)
C8—C9	1.373 (2)	C19—H19	0.93
C8—H8	0.93	C20—O3	1.1915 (19)
C9—H9	0.93	C20—O4	1.3315 (19)
C10—C15	1.381 (2)	C21—O4	1.4415 (19)
C10—C11	1.386 (2)	C21—H21A	0.96
C10—S1	1.7540 (16)	C21—H21B	0.96
C11—C12	1.377 (3)	C21—H21C	0.96
C11—H11	0.93	N1—S1	1.6740 (12)
C12—C13	1.378 (3)	O1—S1	1.4202 (14)
C12—H12	0.93	O2—S1	1.4234 (14)
C13—C14	1.365 (3)		
			
C3—C2—N1	108.20 (13)	C10—C15—H15	120.4
C3—C2—C16	128.09 (15)	C14—C15—H15	120.4
N1—C2—C16	123.67 (14)	C2—C16—H16A	109.5
C2—C3—C4	108.77 (12)	C2—C16—H16B	109.5
C2—C3—C17	126.51 (13)	H16A—C16—H16B	109.5
C4—C3—C17	124.64 (12)	C2—C16—H16C	109.5
C9—C4—C5	119.20 (14)	H16A—C16—H16C	109.5
C9—C4—C3	133.22 (14)	H16B—C16—H16C	109.5
C5—C4—C3	107.57 (13)	C19—C17—C3	118.34 (13)
C6—C5—C4	122.05 (14)	C19—C17—C18	124.36 (14)
C6—C5—N1	130.83 (13)	C3—C17—C18	117.20 (13)
C4—C5—N1	107.12 (13)	C17—C18—H18A	109.5
C7—C6—C5	117.28 (15)	C17—C18—H18B	109.5
C7—C6—H6	121.4	H18A—C18—H18B	109.5
C5—C6—H6	121.4	C17—C18—H18C	109.5
C6—C7—C8	121.55 (17)	H18A—C18—H18C	109.5
C6—C7—H7	119.2	H18B—C18—H18C	109.5
C8—C7—H7	119.2	C17—C19—C20	125.32 (14)
C9—C8—C7	121.06 (17)	C17—C19—H19	117.3
C9—C8—H8	119.5	C20—C19—H19	117.3
C7—C8—H8	119.5	O3—C20—O4	121.87 (14)
C8—C9—C4	118.85 (15)	O3—C20—C19	127.63 (15)
C8—C9—H9	120.6	O4—C20—C19	110.48 (13)
C4—C9—H9	120.6	O4—C21—H21A	109.5
C15—C10—C11	120.80 (16)	O4—C21—H21B	109.5
C15—C10—S1	120.41 (13)	H21A—C21—H21B	109.5
C11—C10—S1	118.76 (12)	O4—C21—H21C	109.5
C12—C11—C10	119.07 (17)	H21A—C21—H21C	109.5
C12—C11—H11	120.5	H21B—C21—H21C	109.5
C10—C11—H11	120.5	C5—N1—C2	108.30 (11)
C11—C12—C13	120.15 (19)	C5—N1—S1	121.12 (10)
C11—C12—H12	119.9	C2—N1—S1	126.50 (11)
C13—C12—H12	119.9	C20—O4—C21	116.60 (13)
C14—C13—C12	120.61 (19)	O1—S1—O2	120.37 (9)
C14—C13—H13	119.7	O1—S1—N1	106.13 (7)
C12—C13—H13	119.7	O2—S1—N1	107.09 (8)
C13—C14—C15	120.24 (18)	O1—S1—C10	108.35 (9)
C13—C14—H14	119.9	O2—S1—C10	108.88 (8)
C15—C14—H14	119.9	N1—S1—C10	104.96 (6)
C10—C15—C14	119.12 (18)		
			
N1—C2—C3—C4	2.26 (16)	C2—C3—C17—C18	60.7 (2)
C16—C2—C3—C4	179.74 (16)	C4—C3—C17—C18	−115.44 (17)
N1—C2—C3—C17	−174.38 (13)	C3—C17—C19—C20	−177.59 (14)
C16—C2—C3—C17	3.1 (3)	C18—C17—C19—C20	−1.3 (3)
C2—C3—C4—C9	179.15 (16)	C17—C19—C20—O3	5.1 (3)
C17—C3—C4—C9	−4.1 (3)	C17—C19—C20—O4	−176.02 (15)
C2—C3—C4—C5	−1.70 (16)	C6—C5—N1—C2	−178.24 (16)
C17—C3—C4—C5	175.01 (13)	C4—C5—N1—C2	0.92 (15)
C9—C4—C5—C6	−1.0 (2)	C6—C5—N1—S1	−19.6 (2)
C3—C4—C5—C6	179.68 (14)	C4—C5—N1—S1	159.59 (10)
C9—C4—C5—N1	179.72 (13)	C3—C2—N1—C5	−2.00 (16)
C3—C4—C5—N1	0.44 (15)	C16—C2—N1—C5	−179.62 (15)
C4—C5—C6—C7	−0.1 (2)	C3—C2—N1—S1	−159.21 (11)
N1—C5—C6—C7	178.98 (16)	C16—C2—N1—S1	23.2 (2)
C5—C6—C7—C8	1.0 (3)	O3—C20—O4—C21	0.4 (3)
C6—C7—C8—C9	−0.9 (3)	C19—C20—O4—C21	−178.62 (15)
C7—C8—C9—C4	−0.3 (3)	C5—N1—S1—O1	50.95 (13)
C5—C4—C9—C8	1.2 (2)	C2—N1—S1—O1	−154.49 (13)
C3—C4—C9—C8	−179.75 (17)	C5—N1—S1—O2	−179.29 (11)
C15—C10—C11—C12	0.4 (2)	C2—N1—S1—O2	−24.73 (14)
S1—C10—C11—C12	−177.57 (14)	C5—N1—S1—C10	−63.66 (12)
C10—C11—C12—C13	0.2 (3)	C2—N1—S1—C10	90.89 (13)
C11—C12—C13—C14	−0.6 (3)	C15—C10—S1—O1	−11.22 (15)
C12—C13—C14—C15	0.6 (3)	C11—C10—S1—O1	166.71 (12)
C11—C10—C15—C14	−0.4 (2)	C15—C10—S1—O2	−143.79 (13)
S1—C10—C15—C14	177.49 (13)	C11—C10—S1—O2	34.14 (14)
C13—C14—C15—C10	−0.1 (3)	C15—C10—S1—N1	101.83 (13)
C2—C3—C17—C19	−122.74 (17)	C11—C10—S1—N1	−80.24 (13)
C4—C3—C17—C19	61.1 (2)		

### Hydrogen-bond geometry (Å, °)

**Table:**

*D*—H···*A*	*D*—H	H···*A*	*D*···*A*	*D*—H···*A*
C6—H6···O2^i^	0.93	2.58	3.391 (2)	146
C13—H13···O3^ii^	0.93	2.50	3.277 (2)	141

Symmetry codes: (i) −*x*+1, *y*−1/2, −*z*+1/2; (ii) *x*−1, *y*−1, *z*.
